# Supplementary material for: Allosteric Neutralization by Human H7N9 Antibodies
Source: Res Sq. 2023 Nov 7:rs.3.rs-3429355. Preprint. [Version 1] doi: 10.21203/rs.3.rs-3429355/v1 (PMC10659534; doi:10.21203/rs.3.rs-3429355/v1)
Supplement: Supplement 1 [file NIHPPRS3429355V1-supplement-1.pdf]

```
Heavy Chain V-gene
           -----------FR1-----------___CDR H1___-----FR2------______CDR H2_____--------------FR3--------------
IGHV4-59   QVQLQESGPGLVKPSETLSLTCTVSGGSIS  SYYWSWIRQPPGKGLEWIGYIYYSGSTN YNPSLKSRVTISVDTSKNQFSLKLSSVTAADTAVYYC
H7.HK1     QVQLQESGPGLVKPSETLSLTCSVSGGSIN  SYYWTWIRQPPGKGLEWVGYIYHSGSTS YNPSLKSRITISVAPSKNHFSLELTSMTAADTAVYYCAR
H7.HK2     QVQLQGSGPGLLRPSETLSLTCSVSGVSIN  SYYWSWVRQPPGKALEWIGYIYYSGNTN YNPSLESRVTISVDRSKNQFSLKMTSVTAADTARYFCAR

IGHV7-4-1  QVQLVQSGSELKKPGASVKVSCKASGYTFT  SYAMNWVRQAPGQGLEWMGWINTNTGNPTYAQGFTGRFVFSLDTSVSTAYLQICSLKAEDTAVYYC
H7.HK3     QVQLVQSGSELKRPGASVKVSCRASGYTFT  SYTINWVRQAPGQGLEWMGWINTSTGDPTYAQGFTGRFVFSLDTSVSTAYLEISRLKAEDTAVYYCAR

IGHV4-61   QVQLQESGPGLVKPSETLSLTCTVSGGSVSSGSYYWSWIRQPPGKGLEWIGYIYYSGSTN YNPSLKSRVTISVDTSKNQFSLKLSSVTAADTAVYYC
H7.HK4     QVQLQESGPGLVKPSETLSLTCTVSGGSVRSASYAWSWIRQPPGKGLEWIGDIYYSGTTN YNPSLKSRVTLSVDTAKNRFSLRLRSVTAADTAVYHCAR

Light Chain V-gene
           -----------FR1---------_____CDR L1_____------FR2------_CDRL2_--------------FR3--------------
IGKV2-28   DIVMTQSPLSLPVTPGEPASISCRSSQSLLHSNGYNYLDWYLQKPGQSPQLLIYLGSNRASGVPDRFSGSGSGTDFTLKISRVEAEDVGVYYC
H7.HK1     DIVMTQSPVSLPVTPGEPASISCNSSQSLLHSNGYAHLDWYLQKPGQSPKLMIYLGLNRAFGVPDRFSGSGSGTDFTLKISRVEAEDVGVYYC
H7.HK2     DIVMTQSPLSLPVTPGEPASISCRSNQSLQHSNGYVHLDWYRQKPGQSPHLLIYLGFNRASGVPDRFSGGGSGTDFTLKISRVEAEDVGVYYC

IGKV1-5    DIQMTQSPSTLSASVGDRVTITCRASQSI   SSWLA    WYQQKPGKAPKLLIYDASSLESGVPSRFSGSGSGTEFTLTISSLQPDDFATYYC
H7.HK3     DIQMTQSPSTLSASVGDRVTITCRASQSI   SSWLA    WYQQKPGKAPKLLIYKASSLESGVPSRFSGSGSGTEFTLTISSLQPDDFATYYC

IGKV1-16   DIQMTQSPSSLSASVGDRVTITCRASQGI   SNYLA    WFQQKPGKAPKSLIYAASSLQSGVPSRFSGSGSGTDFTLTISSLQPEDFATYYC
H7.HK4     DIQMTQSPSSLSASVGDRVTITCRASQGI   RNYLA    WFQQKPGQAPKSLIFAASSLHTGVPSRFSGSGSGTDFTLTISSLQPEDFATYYC

CDR3
           ___CDR H3_____----FR4----      __CDR L3_----FR4---
H7.HK1     LGGHGDYGSDY   WGQGTLVTVSS      MQALQTPFTFGPGTRVDIK
H7.HK2     QGIFGDYGSDY   WGPGTLVTVSS      MQGLQTPFTFGPGTTVDLK
H7.HK3     AFGLTVVRGGIVGVWGQGTTVTVSS      QQYNSYSQTFGQGTKVEIK
H7.HK4     ERYYYGSSGDFDY WGQGTLVTVSS      QHYNSYPPTFGQGTKLEIK
```

Supplementary Fig. 1 H7.HK mAb sequences. Protein sequences of the heavy and light chain variable regions of the H7.HK mAbs are aligned to the putative germline V-genes at top, with amino acid substitutions in red, and in magenta for substitutions shared between the clonally related mAbs H7.HK1 and H7.HK2. Spaces are added to maintain alignment; framework regions (FR) and complementarity-determining regions (CDRs) are indicated based on the Chothia nomenclature. Highlighted in yellow are the mAb residues (paratopes of H7.HK1 and H7.HK2) contacting the H7 antigen. The putative N-linked glycosylation sites on the light chain CDR L1 of H7.HK1 and H7.HK2 and the heavy chain CDR H2 of H7.HK3 are underlined.

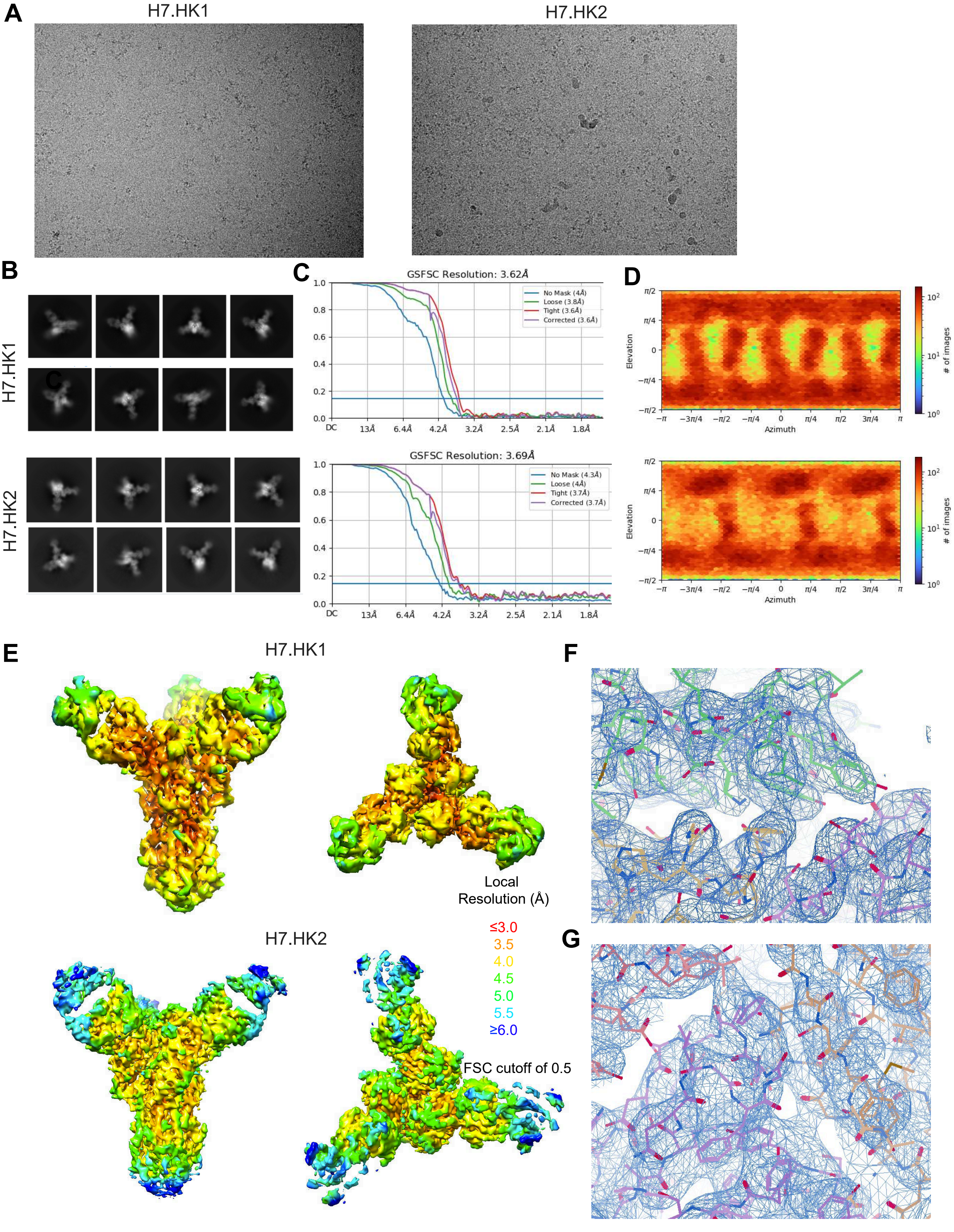
A
H7.HK1
H7.HK2
B
H7.HK1
H7.HK2
C
GSFSC Resolution: 3.62Å
No Mask (4Å)
Loose (3.8Å)
Tight (3.6Å)
Corrected (3.6Å)
GSFSC Resolution: 3.69Å
No Mask (4.3Å)
Loose (4Å)
Tight (3.7Å)
Corrected (3.7Å)
DC
13Å
6.4Å
4.2Å
3.2Å
2.5Å
2.1Å
1.8Å
D
Elevation
Azimuth
# of images
E
H7.HK1
H7.HK2
Local Resolution (Å)
≤3.0
3.5
4.0
4.5
5.0
5.5
≥6.0
FSC cutoff of 0.5
F
G

Supplementary Fig. 2 Cryo-EM details of H7.HK1 and H7.HK2 in complex with H7 SH13 DS2 6R HA trimer. (**A**) Representative micrograph of H7.HK1 (left) and H7.HK2 (right). (**B**) Representative 2D class averages of H7.HK1 and H7.HK2. (**C**) The gold-standard Fourier Shell Correlation (FSC) resulted in a resolution of 3.62 Å for the overall map of H7.HK1 and 3.69 Å for the overall map of H7.HK2. Non-uniform refinement with C3 symmetry was used for both reconstructions. (**D**) The orientations of all particles used in the final refinement are shown as a heatmap. (**E**) The local resolution of the final overall map is shown contoured at 0.0989 for both structures. Resolution estimation was generated through cryoSPARC using an FSC cutoff of 0.5. (**F**) Representative density is shown for the interface of H7.HK1 heavy chain, light chain, and H7 HA. (**G**) Representative density is shown for the interface of H7.HK2 heavy chain, light chain, and H7 HA.

A

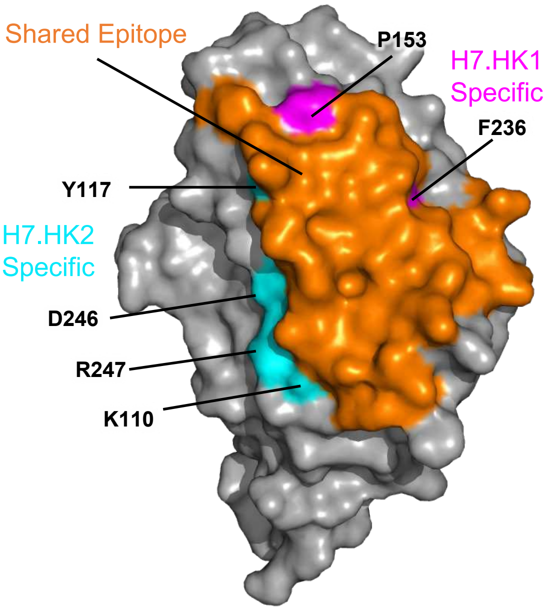

B

	H7.HK1 : **H7**	H7.HK2 : **H7**
Heavy Chain H Bonds	Y52 : **E111**	Y52 : **Y159**
	R97 : **G114**	R97 : **G114**
	G102 : **S158**	G102 : **S158**
	D103 : **T116**	D103 : **T116**
	Y104 : **T156**	Y104 : **T156**
	Y104 : **S158**	Y104 : **S158**
	S106 : **T116**	S106 : **T116**
Heavy Chain Salt Bridge	H53 : **E111**	
Light Chain H Bonds	Y54 : **Q154**	Y54 : **T156**
		S61 : **G119**

C

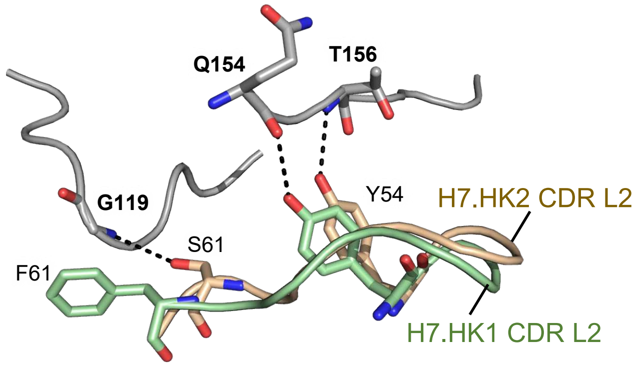

Supplementary Fig. 3 Comparison of H7.HK1 and H7.HK2 binding to H7. (**A**) Differences in epitopes of H7.HK1 and H7.HK2. Majority of surface contacts are conserved, shown in orange. H7.HK1 specific surfaces are shown in magenta, and H7.HK2 specific surfaces are shown in cyan. (**B**) Hydrogen bonds and salt bridges formed by H7.HK1 and H7.HK2 with H7. (**C**) Differences in CDR L2 binding to H7 by H7.HK1 and H7.HK2 as a result of F61S substitution in H7.HK2. S61 forms an additional hydrogen bond with G119 of H7. Additionally, position of Y54 is shifted so that it forms a hydrogen bond with T156 for H7.HK2 instead of Q154 for H7.HK1.

A

```
                                     10        20        30        40        50        60        70        80        90       100
                               ....|....|....|....|....|....|....|....|....|....|....|....|....|....|....|....|....|....|....|....|
Hongkong470129/2013H7N9        DKICLGHHAVSNGTKVNTLTERGVEVVNATETVERTNIPRICSKGKRTVDLGQCGLLGTITGPPQCDQFLEFSADLIIERREGSDVCYPGKFVNEEALRQ
Shanghai2/2013 H7N9            ....................................................................................................
Shanghai4664T/2013 H7N9        ....................................................................................................
Anhui1/2013 H7N9               ....................................................................................................
Zhejiang1/2013 H7N9            ....................................................................................................
Netherlands/219/2003H7N7       .....................................V..............................................................
Guangdong17SF0032016H7N9       .....................................T..............................................................
Hongkong/125/2017 H7N9         ....................................................................................................

                                    110       120       130       140       150       160       170       180       190       200
                               ....|....|....|....|....|....|....|....|....|....|....|....|....|....|....|....|....|....|....|....|
Hongkong470129/2013H7N9        ILRESGGIDKEAMGFTYSGIRANGATSACRRSGSSFYAEMKWLLSNTDNAAFPQMTKSYKNTRKSPALIVWGIHHSVSTAEQTKLYGSGNKLVTVGSSNY
Shanghai2/2013 H7N9            .....................T..............................................................................
Shanghai4664T/2013 H7N9        .....................T..............................................................................
Anhui1/2013 H7N9               .....................T..............................................................................
Zhejiang1/2013 H7N9            .....................T..............................................................................
Netherlands/219/2003H7N7       ...........T.........T..T.......................................D....I......G..T............I.......
Guangdong17SF0032016H7N9       ...........P.....NE..T..V.....................................KE...I................................
Hongkong/125/2017 H7N9         ...........T.....N...T..V....K.....................................I................................

                                    210       220       230       240       250       260       270       280       290       300
                               ....|....|....|....|....|....|....|....|....|....|....|....|....|....|....|....|....|....|....|....|
Hongkong470129/2013H7N9        QQSFVPSPGARPQVNGLSGRIDFHWLMLNPNDTVTFSFNGAFIAPDRASFLRGKSMGIQSGVQVDANCEGDCYHSGGTIISSLPFQNIDSRAVGKCPRYV
Shanghai2/2013 H7N9            .................................................................................N..................
Shanghai4664T2013 H7N9         ........................................................................H........N..................
Anhui1/2013 H7N9               .................................................................................N..................
Zhejiang1/2013 H7N9            .................................................................................N..................
Netherlands/219/2003H7N7       ................Q.........I.................................E....................N......N...........
Guangdong17SF0032016H7N9       ................Q.........I.................................R....................N..................
Hongkong/125/2017 H7N9         ..........................I......................................................N..................

                                    310       320
                               ....|....|....|....|.
Hongkong470129/2013H7N9        KQRSLLLATGMKNVPEIPKGR
Shanghai2/2013 H7N9            .....................
Shanghai4664T2013 H7N9         .....................
Anhui1/2013 H7N9               .....................
Zhejiang1/2013 H7N9            .....................
Netherlands/219/2003H7N7       ..E................R.
Guangdong17SF0032016H7N9       ................V..RK
Hongkong/125/2017 H7N9         .....................
```

B

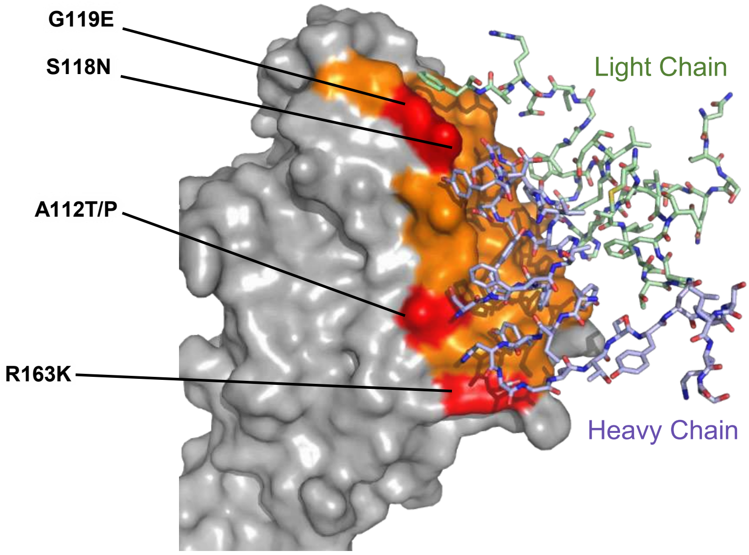

Supplementary Fig. 4 Antigenic drift of H7 HA1 in 2016-2017. (**A**) H7 HA1 protein sequences from the indicated viral isolates are aligned to the 2013 Hong Kong H7N9 autologous isolate at top, with identical amino acids shown in dots. Highlighted in yellow are the H7 residues (epitope) forming contacts with both mAbs H7.HK1 and H7.HK2. H7.HK1 specific epitopes are in magenta; H7.HK2 specific epitopes are in cyan. (**B**) Surface presentation of the H7 HA1 domain highlighting the epitopes (orange) of mAbs H7.HK1 and H7.HK2, with four mutations in red that appeared in the 2016-2017 viral isolates of H7N9. The sticks are interacting CDRs of mAb H7.HK1 heavy and light chains.

Supplementary Table 1 Cryo-EM data collection, refinement, and validation statistics for H7 SH13 DS2 6R HA in complex with H7.HK1 and H7.HK2 Fabs.

	H7 SH13 DS2 6R H7.HK1 (EMD-41422) (PDB: 8TNL)	H7 SH13 DS2 6R H7.HK2 (EMD-41441) (PDB: 8TOA)
**Data collection and processing**		
Magnification	105000	105000
Voltage (kV)	300	300
Electron exposure (e–/Å^2)	58	58
Defocus range (μm)	0.8-2	0.8-2
Pixel size (Å)	0.83	0.83
Symmetry imposed	C3	C3
Initial particle images (no.)	5713957	2339643
Final particle images (no.)	178347	191469
Map resolution (Å)	3.62	3.69
FSC threshold	0.143	0.143
**Refinement**		
Initial model used (PDB code)	6IDD	8TNL
Model resolution (Å)	3.62	3.69
FSC threshold	0.143	0.143
Model composition		
Non-hydrogen atoms	16487	15570
Protein residues	2112	2109
Ligands	7	11
B factors (Å^2)		
Protein	39.71	58.34
Ligand	58.78	48.38
R.m.s. deviations		
Bond lengths (Å)	0.005	0.007
Bond angles (°)	1.121	1.231
Validation		
MolProbity score	1.65	2.23
Clashscore	5.45	12.08
Poor rotamers (%)	0.06	1.62
Ramachandran plot		
Favored (%)	94.86	92.30
Allowed (%)	5.14	7.41
Disallowed (%)	0.0	0.29
